# CITED1 as a marker of favourable outcome in anti-endocrine treated, estrogen-receptor positive, lymph-node negative breast cancer

**DOI:** 10.1186/s13104-023-06376-1

**Published:** 2023-06-15

**Authors:** Malin Dahlgren, Barbara Lettiero, Hina Dalal, Kira Mårtensson, Alexander Gaber, Björn Nodin, Sofia K. Gruvberger-Saal, Lao H. Saal, Jillian Howlin

**Affiliations:** 1 Translational Oncogenomics, Faculty of Medicine, Department of Clinical Sciences Lund and Lund University Cancer Center, Lund University, Lund, Sweden; 2 Therapeutic Pathology, Faculty of Medicine, Department of Clinical Sciences Lund and Lund University Cancer Center, Lund University, Lund, Sweden

**Keywords:** Tamoxifen, CITED1, ERα, Breast cancer, Anti-endocrine, Aromatase inhibitor

## Abstract

**Objective:**

To investigate CITED1 as a potential biomarker of anti-endocrine response and breast cancer recurrence, given its previously determined role in mediating estrogen-dependant transcription. The study is a continuation of earlier work establishing the role of CITED1 in mammary gland development.

**Results:**

*CITED1* mRNA is associated with estrogen-receptor positivity and selectively expressed in the GOBO dataset of cell lines and tumours representing the luminal-molecular subtype. In patients treated with tamoxifen, higher *CITED1* correlated with better outcome, suggesting a role in anti-estrogen response. The effect was particularly evident in the subset of estrogen-receptor positive, lymph-node negative (ER+/LN−) patients although noticeable divergence of the groups was apparent only after five years. Tissue microarray (TMA) analysis further validated the association of CITED1 protein, by immunohistochemistry, with favourable outcome in ER+, tamoxifen-treated patients. Although we also found a favourable response to anti-endocrine treatment in a larger TCGA dataset, the tamoxifen-specific effect was not replicated. Finally, MCF7 cells overexpressing *CITED1* showed selective amplification of *AREG* but not *TGFα* suggesting that maintenance of specific ERα-CITED1 mediated transcription is important for the long-term response to anti-endocrine therapy. These findings together confirm the proposed mechanism of action of CITED1 and support its potential use as a prognostic biomarker.

**Supplementary Information:**

The online version contains supplementary material available at 10.1186/s13104-023-06376-1.

## Introduction

Transcriptional coregulators comprise a family of proteins, which often do not harbour DNA-binding domains but function within complexes to affect target gene expression of a given transcription factor [1]. *CITED1* encodes the founding member of a family of three related proteins with a homologous C-terminal domain responsible for interaction with the coactivator family of P300-CBP [2]. A *CITED1* knockout mouse model we previously described had a mammary gland phenotype defined by stunted ductal outgrowth at puberty and altered transcription of a subset of estrogen-responsive genes. The phenotype could in part be explained by earlier work showing that CITED1 acted as coregulator for the estrogen receptor [3, 4]. We hypothesised that maintenance of the ERα-CITED1 signalling pathway was indicative of a more normally functioning epithelium and subsequently showed that *CITED1* correlated with better breast cancer prognosis in a tumour dataset [5]. In this study we sought to verify its proposed function as an ERα-coregulator specifically in breast cancer cells, further elucidate its potential role as a prognostic indicator, and investigate its relevance, if any, for treatment response. To do this, we examined *CITED1* mRNA and protein expression, in human breast cancer cell lines as well as several independent tumour datasets. Interrogating the publicly available database, GOBO, served as a starting point to develop our hypotheses. To further validate our observations, we used a previously described tissue microarray (TMA) and the publicly available TCGA breast cancer dataset. We additionally generated MCF7 cells stably overexpressing *CITED1* to examine changes in gene transcription.

## Main text

### Results and discussion

#### CITED1 is expressed in the breast cancer cell lines and tumours of the ER+/luminal subtype and correlates with better prognosis in a tamoxifen-treated cohort

**Fig. 1:**
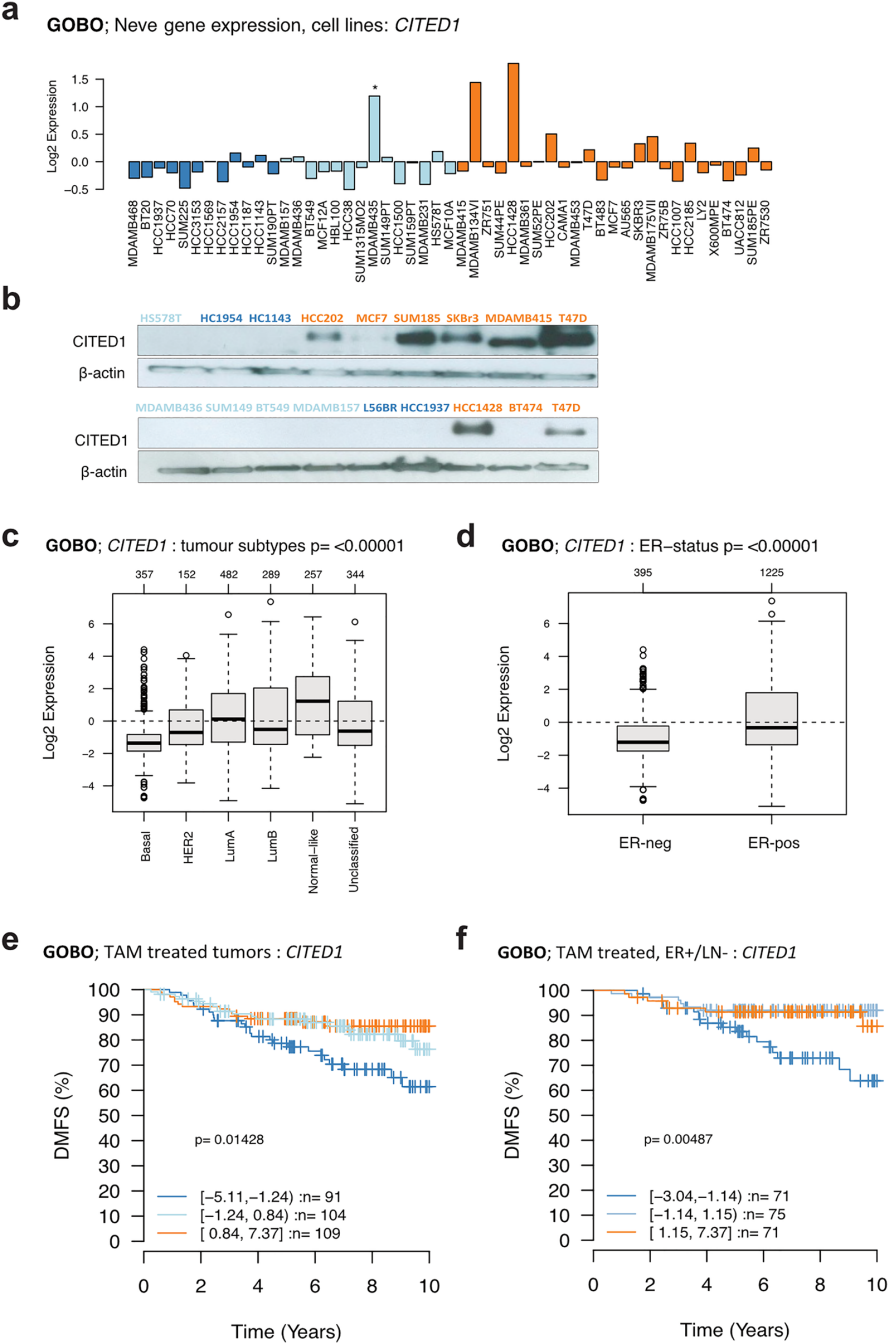
*CITED1* is expressed in cell lines of the ER+ luminal breast cancer subtype and correlates with tamoxifen response in vivo. **A** GOBO database of breast cancer cell lines showing relative expression of *CITED1* mRNA ordered by tumour molecular subtypes representation. Luminal—orange, Basal A—light blue, Basal B—blue *Misclassified melanoma cell line, MDAMB435 [36]. **B** CITED1 protein expression is shown in breast cancer cell lines representing the different tumour molecular subtypes. β-actin is used as a loading control. **C** *CITED1* expression in the various molecular subtypes represented in the GOBO breast tumour dataset. **D** *CITED1* expression in ER+ and ER− tumours in the GOBO tumour dataset. **E** Survival analysis showing distant metastasis-free survival (DMFS) in patients with breast cancer treated with tamoxifen (TAM). Patients were classified into 3 groups according to expression: high *CITED1*—orange, medium *CITED1*—light blue, low *CITED1*—blue. **F** Survival analysis for the cohort of ER+/LN− patients treated with tamoxifen (TAM). The number of patients in each group at diagnosis is indicated as *n*

The GOBO database allows for the investigation of gene expression across a panel of 51 breast cancer cell lines and 1881 breast tumours with the advantage of stratifying by molecular subtype [6]. The molecular subtypes (ER+/luminal, HER2, and basal-like) established in a landmark paper in 2000, redefined our understanding of breast cancer in terms of a tumour genotype/phenotype that could explain differences in clinical presentation and treatment response, and inform prognosis [7, 8]. Interrogation of this database revealed that *CITED1* is expressed in cell lines that represent the ER+/luminal subtypes (MCF, T47D, SkBr3) but absent in basal-like cell lines (HCC1937, BT549, Sum149) (Fig. 1a). Western blot analysis confirmed that this correlation extended to protein expression (Fig. 1b). Moreover, the association with the luminal subtype was retained in tumours, and our previous observation that *CITED1* expression correlated with ERα-positivity was confirmed (Fig. 1c, d) [5, 9, 10]. Further analysis revealed that patients treated with tamoxifen (TAM) with high relative *CITED1* expression, had increased distant metastasis-free survival (DMFS) (Fig. 1e). This was not simply reflecting ERα gene expression as *ESR1* alone was not prognostic in this dataset. Furthermore, the survival rate of patients in the low *CITED1* expression group were comparable to the untreated group (Additional file 1: Fig. S1a, b). The difference in DMFS was especially apparent in the subset of tamoxifen-treated tumours that were estrogen-receptor positive, lymph node negative (ER+/LN−). (Fig. 1f). This observation is notable because these are typically considered a good prognostic group where, in most cases, surgery, with or without adjuvant treatment, is curative. Patients can however succumb to much later recurrence, even as much as 20 years after their primary cancer. The absolute risk for low-grade, LN− tumours (T1N0) is approximately 10% in the 5–20 years following diagnosis [11]. Having means to identify and parse high-risk/non-responders and specifically later recurrence is therefore highly sought after.

#### CITED1 protein expression in tamoxifen-treated tumours correlates with better patient outcome

**Fig. 2:**
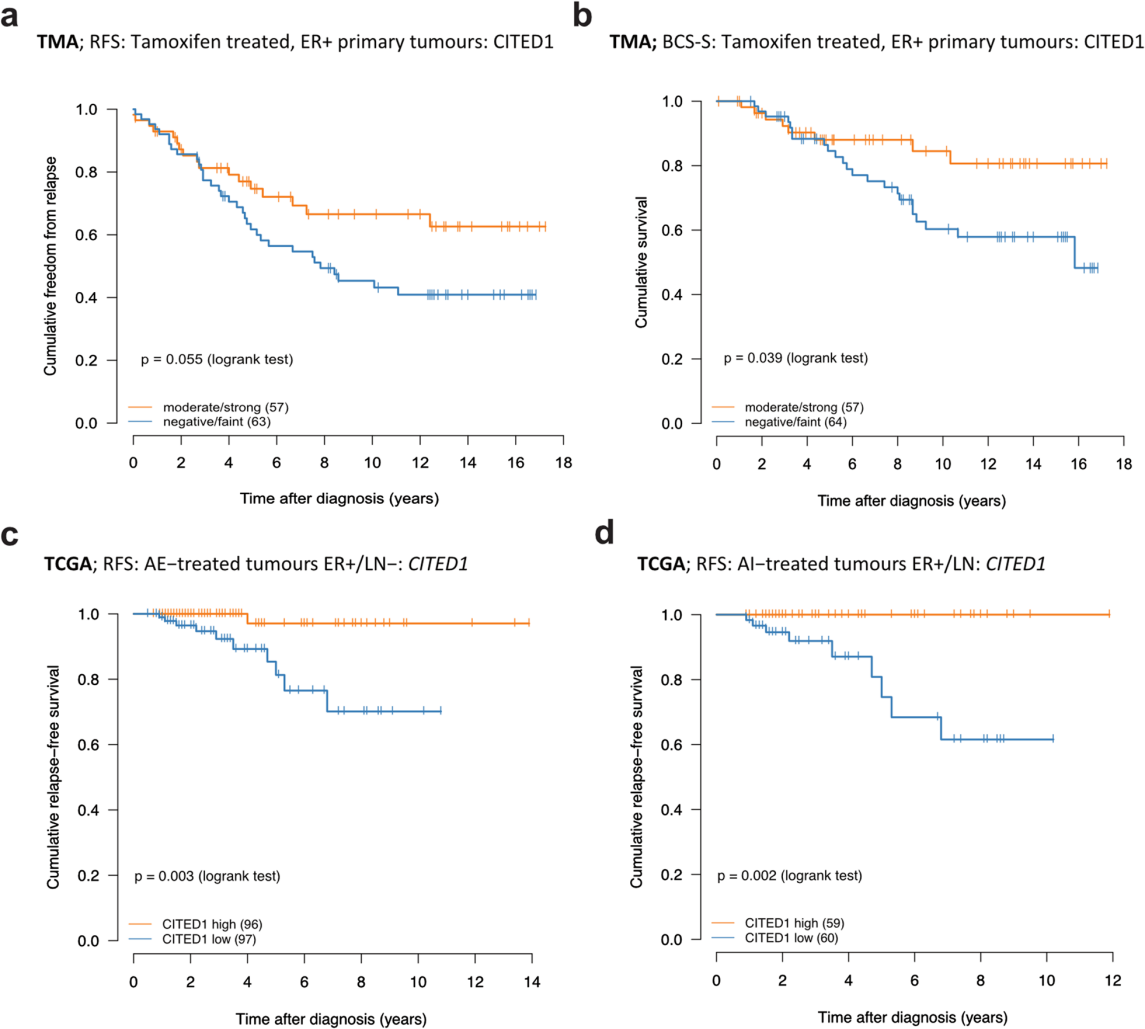
Correlation of CITED1 protein (TMA) and gene expression (TGCA) with prognosis following anti-endocrine treatment. For the TMA analysis, patients were classified into 2 groups either as having low (negative/faint: blue line) or high (moderate/strong: orange line) CITED1 protein expression following IHC; **A** Relapse-free survival (RFS) following breast cancer diagnosis in the ER+ , TAM-treated cohort. **B** Breast cancer specific survival (BCSS) following diagnosis in the ER+ , TAM-treated cohort. The number of patients in each group at diagnosis is indicated in brackets. TCGA was used for validation of differential *CITED1* gene expression (high expression: orange line, low expression: blue line) and its association with prognosis; **C** Relapse-free survival in all ER+/LN− TCGA breast tumours following any anti-endocrine (AE) treatment. **D** Relapse-free survival in the ER+/LN− breast tumours treated with aromatase inhibitors (AI). The number of patients in each group at diagnosis is indicated in brackets

Following surgery, tumours are processed using routine clinical pathology to assess grade, receptor expression and tumour subtype that informs treatment and prognosis. A biomarker that could be added to this standard analysis would be a valuable addition to current practice [12]. We validated the specificity of the CITED1 antibody previously [13, 14] and a TMA analysis served both to evaluate the feasibility of immunohistochemistry and to determine if the correlation seen with *CITED1* mRNA and favourable prognosis was sustained. The TMA has been previously described and consists of approximately 400 breast tumours with long term follow up [15]. Using comparable parameters to the GOBO analysis (tamoxifen-treated/ER+) we found that relapse-free (RFS) and disease-specific survival (BCSS) was significantly better for patients with higher expression of CITED1 (Fig. 2a, b). Examining tamoxifen-treated, ER+/LN− yielded very few patients although the difference in survival was still apparent (Additional file 1: Fig. S1c). As with GOBO, no difference in survival was seen in the tamoxifen-untreated tumours (Additional file 1: Fig. S1d).

#### A CITED1 anti-endocrine but not tamoxifen-specific effect is replicated in the TCGA ER+/LN- tumours

We used TCGA to try to validate our findings using more contemporary data. The TCGA breast tumours represent patients with 2009 as their median year of diagnosis (range: 1988–2013) and includes several anti-endocrine (AE) treatments (56% aromatase inhibitors (AI), 42% tamoxifen). We failed to see an association in tamoxifen-only treated patients (Additional file 1: Fig. S1e) but *CITED1* expression was significantly associated with favourable outcome when we extended the analysis to include all AE, or AI-only treatment, specifically in the ER+/LN− cohort (Fig. 2c, d). This underscored the ability of *CITED1* to risk-stratify patients receiving AE-treatment in a third independent dataset. One obvious difference between the datasets, which may in part explain the discrepancy, is that 71% of the TAM-treated tumours in the GOBO data were ER+/LN− at diagnosis, compared with only 35% of the TCGA TMA-treated tumours. Additionally, very few events occurred in the TCGA TAM-treated ER+/LN− cohort (Additional file 1: Fig. S1f). Furthermore, GOBO patients received a mean 2 years (range: 1–7) of treatment, as opposed to the current guideline recommendation of 5, with extension up to 10 years [16, 17]

If AI-treatment response is affected, this suggests a role for ERα-CITED1 complex formation even in an estrogen-depleted environment. The availability of CITED1 may function to oppose mechanisms of AI resistance that disrupt normal ERα-signalling or trigger ligand-independent activation. Induction of ERα adaptive hypersensitivity to residual estrogen by AE/AI treatment is thought to be mediated by various growth factor signalling pathways, which in turn can alter the genome-wide binding pattern of ERα via posttranslational modifications. This implies the availability of specific co-factors may be of paramount importance to hold aberrant ERα-signalling in check [18].

#### Selective CITED1-dependant AREG expression in MCF7 breast cancer cells

**Fig. 3:**
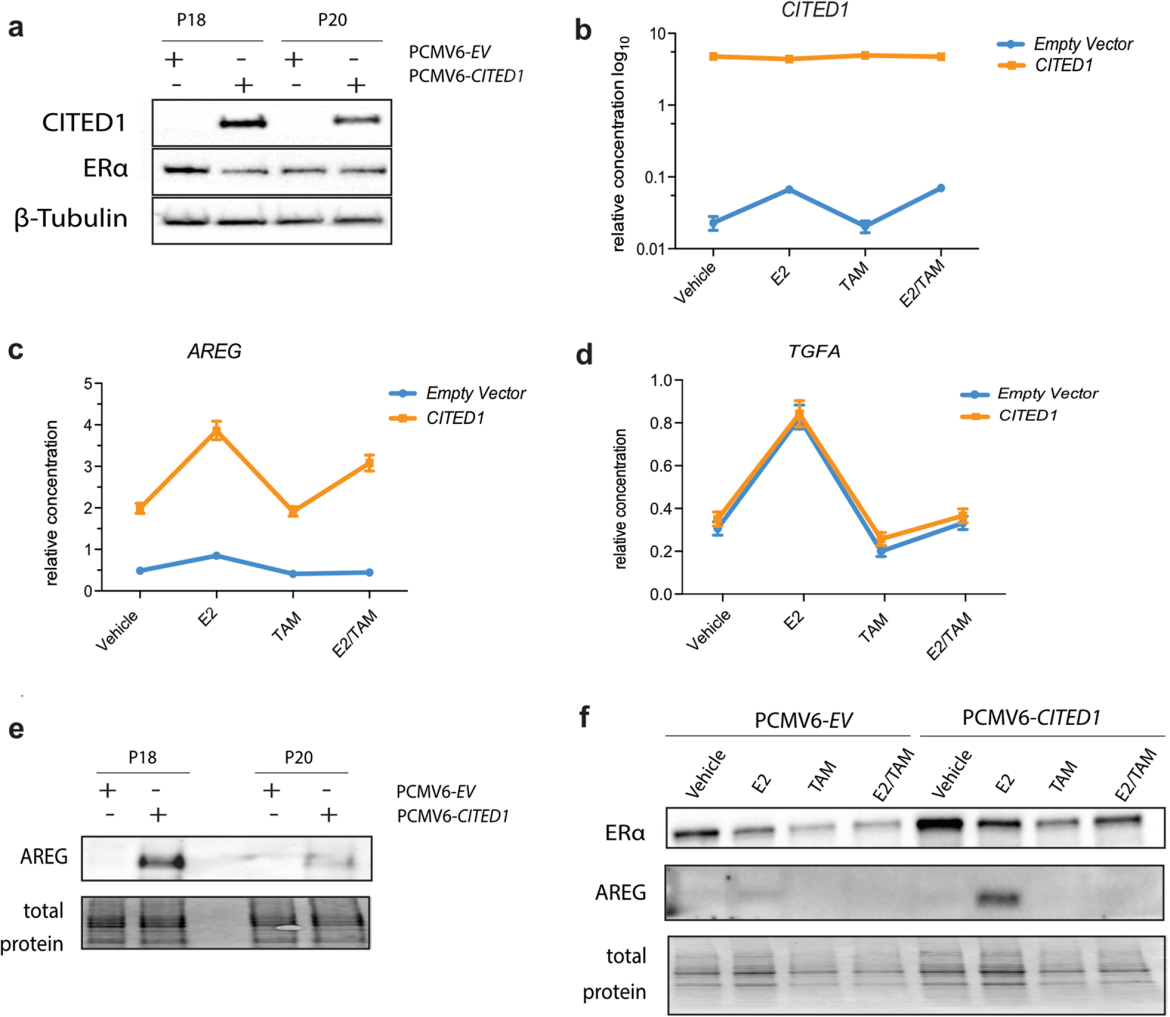
CITED1 overexpression alters expression of amphiregulin. **A** Characterization of CITED1 (28 kDa) and ERα (66KDa) protein expression in stable *CITED1*-overexpressing MCF7 cells compared to the empty vector (EV) control following passage (P) under selection (G418 antibiotic). β-tubulin (55 kDa) is used as a loading control. **B** *CITED1* concentration, relative to a *IPO8* control, in stable *CITED1*-overexpressing MCF7 cells (orange) compared to the empty vector control (blue) in response to estrogen stimulation (E2), tamoxifen (TAM) or simultaneous (E2/TAM) treatment. **C**, **D** Concentration of *AREG* and *TGFα* relative to a *IPO8* control is shown in stable *CITED1*-overexpressing MCF7 cells (orange) compared to the stable empty vector control (blue). The response to estrogen stimulation (E2), tamoxifen (TAM) or simultaneous (E2/TAM) treatment is shown. **E** AREG protein expression in stable *CITED1*-overexpressing MCF7 cells compared to the empty vector (EV) control following passage under selection. The most intense band signal for AREG was just over the 25KDa marker which would likely correspond to the previously reported 26-28KDa cell surface form [30]. Total protein staining is used as a loading control. **F** AREG protein expression in stable *CITED1*-overexpressing MCF7 cells compared to the empty vector (EV) control in response to estrogen stimulation (E2), tamoxifen (TAM) or simultaneous (E2/TAM) treatment. Total protein staining is used as a loading control

In the murine mammary gland, gene expression profiling of the *CITED1*-knockout mouse revealed dysregulation of several ERα-responsive genes [3]. A major driver of the process of pubertal mammary epithelial outgrowth, downstream of ERα-CITED1, is the EGFR-ligand, amphiregulin (*AREG*) [3, 19]. *AREG* is selectively overexpressed at the initiation of pubertal expansion and reduced in the *CITED1*-null mammary epithelium concomitant with retarded ductal outgrowth [3, 5]. Furthermore, *AREG* has been identified as a downstream effector of estrogen in ERα+ breast cancer and its expression is necessary for the growth of MCF7 xenografts [20]. We wanted to examine if ERα-*AREG* signalling is also CITED1-mediated in breast cancer cells and the relatively low levels of CITED1 in MCF7s compared to other luminal-types made them ideal for development of a *CITED1*-overexpression model (Figs. 1b, 3a, b). We found increased expression of AREG, mRNA and protein, in MCF7 cells stably overexpressing *CITED1*; both basal expression and transcriptional response to estrogen treatment (Fig. 3c, e, f). In agreement with our previous observations, manipulation of *CITED1* levels did not simply lead to a general enhancement of transcription of estrogen responsive genes as evidence by the lack of effect on *TGFα* (Fig. 3d) [3]. These results serve as a proof-of-concept that selective signalling pathways driven by ERα-CITED1 in human breast cancer cells mirror those driving murine mammary gland outgrowth. AREG has been previously associated with AI-resistance in vitro due to growth inhibition escape by activation of EGFR [21]. We hypothesise however, that in luminal-type/ER+/LN− breast cancer, *AREG* expression downstream of ERα-CITED1 may be associated with an active ERα-coregulator complex that is more amenable to endocrine antagonism. We theorise that an active ERα-CITED1-AREG response acts a surrogate for intrinsic estrogen and AE sensitivity and that CITED1-mediated ERα-transcription is central to the AE response.

Targeting coregulators in disease has for many years been seen as attractive due to the potential advantage of altering specific transcriptional programmes while leaving other key signalling nodes intact[22–26]. The potential for future therapeutic targeting of CITED1 is further strengthened by recent developments of co-regulator interference/manipulation by peptides, such as a PELP-derived peptide targeting ERα signalling and a CITED2-derived peptide which interferes with HIF1α/p300 interaction [27, 28].

### Limitations

- Interrogation of newer, larger datasets and prospective studies will be required to evaluate if CITED1 can provide unique or additional diagnostic or prognostic information.
- We have not examined the role of (neo-)adjuvant chemotherapy in the low-risk group identified by *CITED1*. Given the more widespread use of prediction tools to limit unnecessary chemotherapy (Oncotype DX, MammaPrint etc.) it is of interest to investigate if CITED1 expression may identify those patients that would benefit less from more aggressive, extended, or consecutive AE treatments.
- We used the differential response of *AREG* and *TGFα* to confirm our hypothesis that CITED1 could affect ERα target genes in human breast cancer cells. A more extensive/transcriptome-wide analysis would provide the possibility to select for a gene set reflecting a prognostically favourable active ERα-CITED1-driven expression signature and expand the biomarker potential of our observation [29].
- We have not explored and cannot accurately define, the functional significance of the AREG protein downstream of ERα-CITED1 more than to say its expression is increased. We detected increased levels of a ~ 28KDa form, likely representing a cell surface N-terminal proteolytic cleavage product. However, the functional significance of this is unclear as several cell-surface and soluble forms can result from the processing of the AREG pre-protein to the cleaved biologically active ligand [30]. Further investigation is outside the scope of this study but the importance of AREG protein cleavage has been addressed elsewhere [31, 32].

### Methods

#### Cell lines

Cells were obtained from ATCC and grown in accordance with recommendations. Transfections were performed using Lipofectamine2000 and Opti-MEM reduced serum media (Life Technologies). *CITED1* was overexpressed using a pCMV6 containing human *CITED1*, transcript isoform 1 (pCMV6-*CITED1*, Origene, #PS100001). An empty vector, CMV6 expression plasmid was used as the negative control (pCMV-*EV*, Origene, #RC202419). Stable cell lines were established through antibiotic selection using G418 1 mg/ml (A1720 Sigma-Aldrich).

#### Drug treatment

β-E_2_(#E8875, Sigma) was used at 10 nM–100 nM, TAM (#H7904, Sigma) was used at 1 µM. Before 3 h drug stimulation, cells were starved for 24 h in phenol-red free medium containing 5% charcoal-treated FBS (HyClone). Cells were lysed with BufferRLTplus (RNeasy®plus Mini Kit, Qiagen), supplemented with 1% β-mercaptoethanol for subsequent RNA isolation. Whole cell lysates for protein analysis were taken in parallel.

#### Immunoblotting and IHC

Anti-CITED1 (rabbit, #AB15096, Abcam); anti-ERα (rabbit, #8644, Cell Signalling); anti-ERα (mouse, Abcam), anti-β-Actin (AC-15), #A5441 from Sigma-Aldrich, anti-Amphiregulin (goat, R&D Systems #AF262). Lysates were resolved by SDS-PAGE (4–20% pre-cast, stain-free, Bio-Rad) and transferred to PVDF membranes. The membranes were blocked (5% non-fat milk/TBST) prior to incubation with diluted primary antibodies (2.5% non-fat milk/ TBST). The blots were probed with appropriate diluted secondary antibodies (5% non-fat milk/TBST) (Pierce Biotechnology). Membranes were cut according to size prior to antibody incubation but where proteins had similar molecular weights, blots were run separately (Additional file 2). Membranes were developed using ECL (Bio-Rad). The TMA was processed as previously described [15]; A 2-group combined CITED1 scoring system reflecting cytoplasmic staining fraction and staining intensity was used.

#### Data analysis and statistics

Data analysis was performed using R v 4.2.1 with relevant packages; see https://github.com/CoregulomicsUnit/CITED1_BC for further details on data extraction and processing. Briefly, gene expression and clinical data from ProjectID: TCGA-BRCA were downloaded from https://portal.gdc.cancer.gov, accessed using TCGABiolinks [33]. Log transformed, batch corrected (using TCGAbatch_Correction function) gene expression data log2(TPM + 0.1) was used for survival analysis [34]. TCGA-BRCA patients were stratified into two groups based on *CITED1* expression (cut-off: median). The same survival package was used for analysis of both TMA and TCGA cohorts; p-values were determined using the logrank test [35].

#### Droplet digital PCR

RNA was isolated using Qiagen RNeasy Plus mini-kit and quantified using Nanodrop (ThermoScientific). cDNA was generated using ‘iScript Advanced cDNA synthesis for RT-qPCR’ (Bio-Rad). Bio-Rad’s ‘ddPCR Supermix for Probes’ was used with predesigned TaqMan assays (Applied Biosystems) consisting of specific primers and FAM/VIC labelled probes for *CITED1* (#Hs00918445_g1), *IPO8* (#Hs00183533_m1), *AREG* (Hs00950669_m1), *TGFα* (Hs00608187_m1); run on the Bio-Rad QX200 instrument. A manual cut-off for positive droplets was selected using the Bio-Rad QuantaSoft™ data analysis suite to calculate the copies/µl of each transcript relative to the internal *IPO8* control.

## Supplementary Information

**Additional file 1: Figure S1.** **A** Expression of the estrogen receptor gene, *ESR1*, does not determine outcome (DMFS) in the subset of tamoxifen (TAM) treated ER + /LN- tumours in the GOBO dataset. **B** *CITED1* expression in the cohort of untreated tumours (No TAM) in the GOBO dataset is not associated with better prognosis. Patients are classified into 3 groups according to expression (high *CITED1*—orange, medium *CITED1*—light blue, low *CITED1*—blue) and the number of patients in each group at diagnosis is indicated as *n*. **C** CITED1 protein expression is not associated with survival in untreated (No TAM) tumours in the TMA dataset. **D** CITED1 protein expression is associated with increased survival in the ER+/LN−tamoxifen (TAM) treated cohort of TMA tumours. Patients are classified into 2 groups either as having low (negative/faint: blue line) or high (moderate/strong: orange line) CITED1 protein expression following IHC and the number of patients in each group at diagnosis is indicated in brackets. **E** *CITED1* expression in TCGA breast cancer dataset is not associated with increased relapse-free survival in tamoxifen (TAM) treated tumours. **F** *CITED1* expression in TCGA breast cancer dataset is not associated with increased relapse-free survival in ER+/LN− tamoxifen (TAM) treated tumours. Patients are classified into 2 groups (high expression: orange line, low expression: blue line) and the number of patients in each group at diagnosis is indicated in brackets.

**Additional file 2: Figure S2.** Extended and uncropped blot images, ladder markers and total protein controls. **A** Uncropped images of the CITED1 blots in Fig. 1b showing the edges of the X-ray film. **B** The molecular weight markers routinely used in the lab and the standard cutting pattern of the membranes prior to incubation with antibodies. **C** Extended and overexposed images of the blots in Fig. 3a. Due to the strong ECL signal even long exposure did not reveal other bands. The membrane edges are visible only with extreme over exposure for CITED1, ER-α and β-tubulin. An additional protein loading control is provided. Note that only the first 4 lanes are shown in Fig. 3a. **D** Extended image of the AREG blot in Fig. 3e with ladder bands visible. An extended protein loading control is also shown with visible ladder markers. **E** Extended image of the AREG and ER-α blots in Fig. 3f. For ER-α, a long exposure is provided as well as an image of the membrane whole molecular weight markers to illustrate the size of the cut membrane. The molecular weight ladder bands are visible on the AREG blot. An extended protein loading control is also shown with visible ladder markers.

## Data Availability

The cell lines, plasmids, antibodies and drugs used in experiments were commercially purchased. GOBO data is accessible at http://co.bmc.lu.se/gobo/gsa_cellines.pl. TCGA data is available at https://portal.gdc.cancer.gov. The TMA data has been previously described [15] R-code used in the various analyses can be found at https://github.com/CoregulomicsUnit/CITED1_BC.

## Abbreviations

P300: Previously EP300
CBP: Previously CREB-binding protein
ER: Estrogen receptor
ERα: Estrogen receptor alpha
ESR1: Estrogen receptor alpha gene
LN: Lymph node
TGFα: Transforming growth factor alpha
AREG: Amphiregulin
TAM: Tamoxifen
TMA: Tissue microarray
IHC: Immunohistochemistry
GOBO: Gene Expression Based Outcome for Breast Cancer Online
TCGA: The Cancer Genome Atlas
AE: Anti-endocrine
AI: Aromatase inhibitor
