# Supplementary figures and images for: CITED1 as a marker of favourable outcome in anti-endocrine treated, estrogen-receptor positive, lymph-node negative breast cancer
Source: BMC Res Notes. 2023 Jun 15;16:105. doi: 10.1186/s13104-023-06376-1 (PMC10268435; doi:10.1186/s13104-023-06376-1)

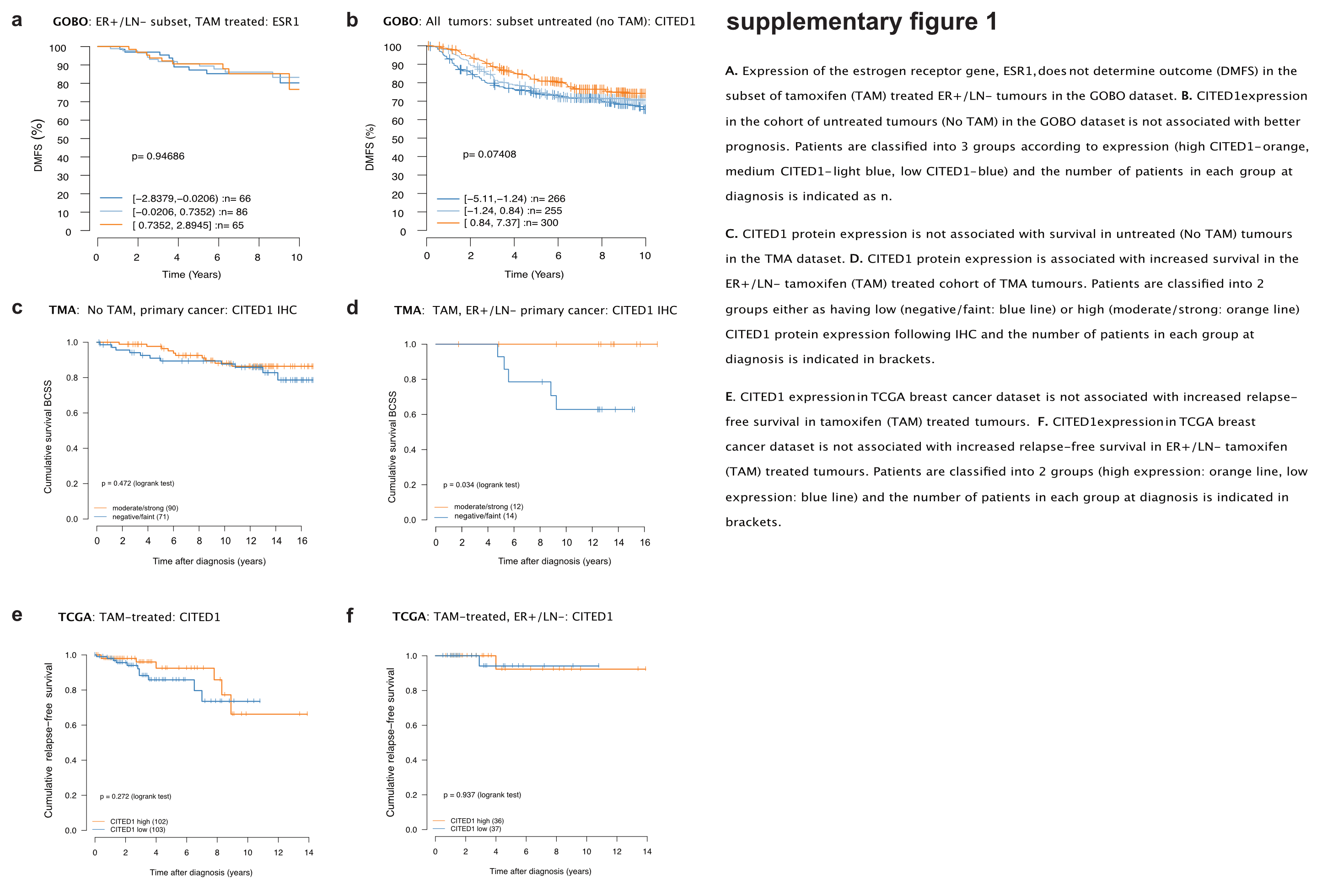

Supplement: Supplementary file 1 — Additional file 1: Figure S1. A Expression of the estrogen receptor gene, ESR1, does not determine outcome (DMFS) in the subset of tamoxifen (TAM) treated ER + /LN- tumours in the GOBO dataset. B CITED1 expression in the cohort of untreated tumours (No TAM) in the GOBO dataset is not associated with better prognosis. Patients are classified into 3 groups according to expression (high CITED1—orange, medium CITED1—light blue, low CITED1—blue) and the number of patients in each group at diagnosis is indicated as n. C CITED1 protein expression is not associated with survival in untreated (No TAM) tumours in the TMA dataset. D CITED1 protein expression is associated with increased survival in the ER+/LN−tamoxifen (TAM) treated cohort of TMA tumours. Patients are classified into 2 groups either as having low (negative/faint: blue line) or high (moderate/strong: orange line) CITED1 protein expression following IHC and the number of patients in each group at diagnosis is indicated in brackets. E CITED1 expression in TCGA breast cancer dataset is not associated with increased relapse-free survival in tamoxifen (TAM) treated tumours. F CITED1 expression in TCGA breast cancer dataset is not associated with increased relapse-free survival in ER+/LN− tamoxifen (TAM) treated tumours. Patients are classified into 2 groups (high expression: orange line, low expression: blue line) and the number of patients in each group at diagnosis is indicated in brackets. [file 13104_2023_6376_MOESM1_ESM.png]

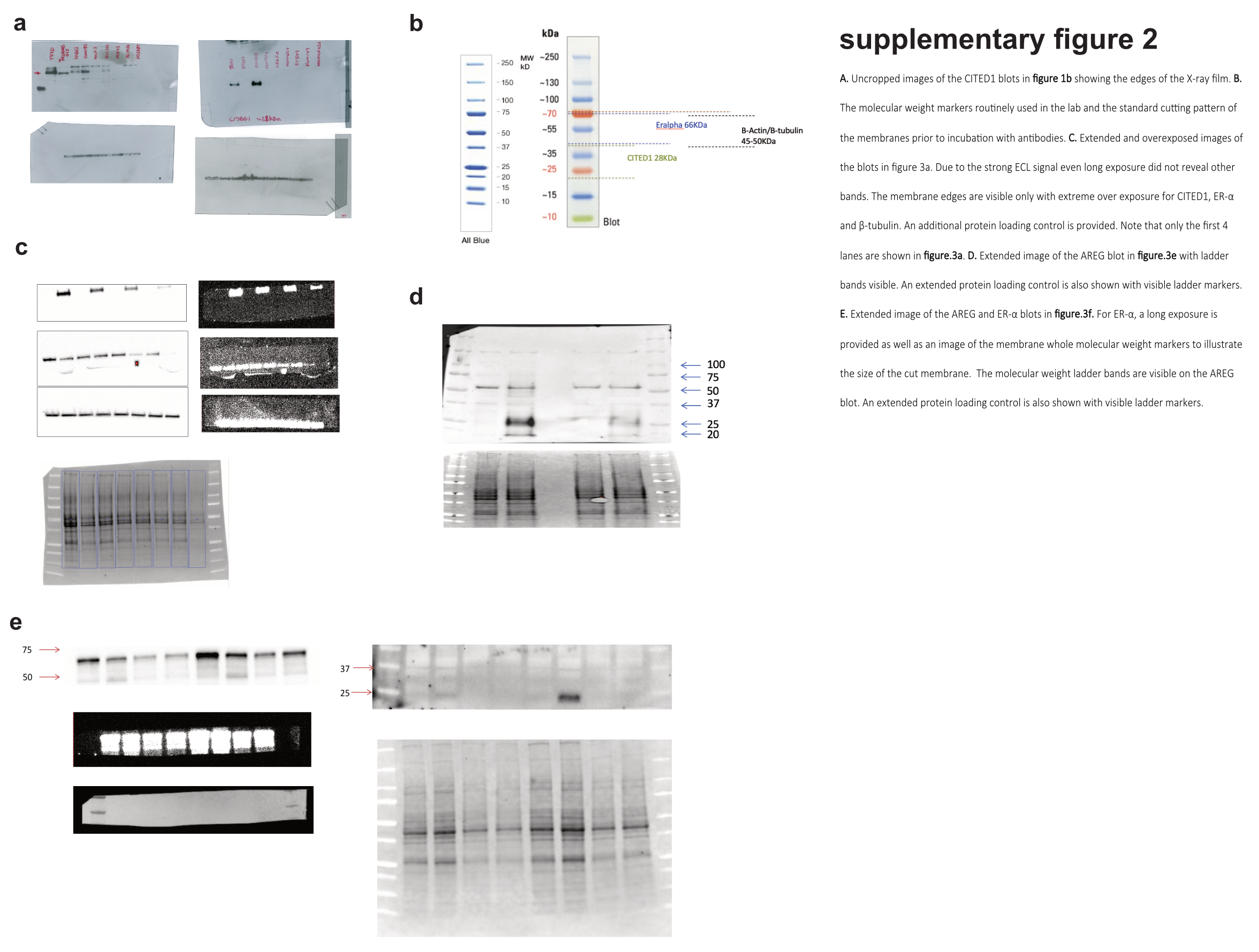

Supplement: Supplementary file 2 — Additional file 2: Figure S2. Extended and uncropped blot images, ladder markers and total protein controls. A Uncropped images of the CITED1 blots in Fig. 1b showing the edges of the X-ray film. B The molecular weight markers routinely used in the lab and the standard cutting pattern of the membranes prior to incubation with antibodies. C Extended and overexposed images of the blots in Fig. 3a. Due to the strong ECL signal even long exposure did not reveal other bands. The membrane edges are visible only with extreme over exposure for CITED1, ER-α and β-tubulin. An additional protein loading control is provided. Note that only the first 4 lanes are shown in Fig. 3a. D Extended image of the AREG blot in Fig. 3e with ladder bands visible. An extended protein loading control is also shown with visible ladder markers. E Extended image of the AREG and ER-α blots in Fig. 3f. For ER-α, a long exposure is provided as well as an image of the membrane whole molecular weight markers to illustrate the size of the cut membrane. The molecular weight ladder bands are visible on the AREG blot. An extended protein loading control is also shown with visible ladder markers. [file 13104_2023_6376_MOESM2_ESM.png]
